# A murine model for the del(*GJB6*-D13S1830) deletion recapitulating the phenotype of human DFNB1 hearing impairment: generation and functional and histopathological study

**DOI:** 10.1186/s12864-024-10289-z

**Published:** 2024-04-11

**Authors:** María Domínguez-Ruiz, Silvia Murillo-Cuesta, Julio Contreras, Marta Cantero, Gema Garrido, Belén Martín-Bernardo, Elena Gómez-Rosas, Almudena Fernández, Francisco J. del Castillo, Lluís Montoliu, Isabel Varela-Nieto, Ignacio del Castillo

**Affiliations:** 1 Servicio de Genética, Hospital Universitario Ramón y Cajal, IRYCIS, Madrid, Spain; 2 Institute for Biomedical Research “Sols-Morreale”, Spanish National Research Council-Autonomous University of Madrid, Madrid, Spain; 3 Hospital La Paz Institute for Health Research (IdiPAZ), Madrid, Spain; 4 Anatomy and Embryology Department, Faculty of Veterinary, Universidad Complutense de Madrid, Madrid, Spain; 5 Department of Molecular and Cellular Biology, National Centre for Biotechnology (CNB-CSIC), Madrid, Spain; 6 Centro de Investigación Biomédica en Red de Enfermedades Raras (CIBERER-ISCIII), Madrid, Spain

**Keywords:** Inherited hearing impairment, DFNB1, *GJB2*, Connnexin-26, Murine models

## Abstract

Inherited hearing impairment is a remarkably heterogeneous monogenic condition, involving hundreds of genes, most of them with very small (< 1%) epidemiological contributions. The exception is *GJB2*, the gene encoding connexin-26 and underlying DFNB1, which is the most frequent type of autosomal recessive non-syndromic hearing impairment (ARNSHI) in most populations (up to 40% of ARNSHI cases). DFNB1 is caused by different types of pathogenic variants in *GJB2*, but also by large deletions that keep the gene intact but remove an upstream regulatory element that is essential for its expression. Such large deletions, found in most populations, behave as complete loss-of-function variants, usually associated with a profound hearing impairment. By using CRISPR-Cas9 genetic edition, we have generated a murine model (*Dfnb1*^*em274*^) that reproduces the most frequent of those deletions, del(*GJB6*-D13S1830). *Dfnb1*^*em274*^ homozygous mice are viable, bypassing the embryonic lethality of the *Gjb2* knockout, and present a phenotype of profound hearing loss (> 90 dB SPL) that correlates with specific structural abnormalities in the cochlea. We show that *Gjb2* expression is nearly abolished and its protein product, Cx26, is nearly absent all throughout the cochlea, unlike previous conditional knockouts in which *Gjb2* ablation was not obtained in all cell types. The *Dfnb1*^*em274*^ model recapitulates the clinical presentation of patients harbouring the del(*GJB6*-D13S1830) variant and thus it is a valuable tool to study the pathological mechanisms of DFNB1 and to assay therapies for this most frequent type of human ARNSHI.

**Supplementary Information:**

The online version contains supplementary material available at 10.1186/s12864-024-10289-z.

## Introduction

Inherited hearing impairment is a very heterogeneous group of disorders, which differ in their clinical features and genetic causes. Hearing loss can manifest as an isolated condition (non-syndromic hearing impairment, NSHI) or in association with clinical signs in other organs, as it occurs in hundreds of syndromes. Likewise, hundreds of genes are involved in the syndromic conditions, and over 130 genes in NSHI. All patterns of inheritance have been reported, with a predominance of autosomal recessive forms [38]. Given this extreme heterogeneity, most of the different subtypes of inherited hearing impairment have a very small epidemiological contribution to the whole [19]. A remarkable exception is DFNB1, which is by far the most frequent type in most studied populations (up to 40% of all cases of autosomal recessive non-syndromic hearing impairment, ARNSHI) [8].

DFNB1 ARNSHI is caused by pathogenic variants that affect the *GJB2* gene, which encodes connexin-26 (Cx26), a protein of the intercellular gap junctions [27]. *GJB2* is expressed in diverse cell types from many different tissues. In the inner ear, Cx26 is a component of two gap junction networks: the epithelial network, which couples all the supporting cells of the organ of Corti, interdental cells of the spiral limbus, and root cells in the spiral ligament; and the connective tissue network, which couples fibrocytes and mesenchymal cells in the lateral wall, and basal and intermediate cells of the stria vascularis. Cx26 is also expressed in type I neurons of the spiral ganglion [30]. Of note, it is not expressed in the sensory hair cells. Connexin channels in these gap junction networks play a variety of roles: i) spatial buffering of potassium ions, which, after intervening in the transduction of the auditory signal, need to be dispersed through the gap junction networks to avoid the toxic effects of their accumulation [24],ii) transport of metabolites, especially glucose [9],iii) transport of potassium ions within the stria vascularis for their secretion into endolymph [39], iv) intercellular transport of inositol 1,4,5-trisphosphate (IP3) and propagation of the subsequent calcium waves, which coordinate the activity of the supporting cells [3]. In addition, Cx26 hemichannels also participate in purinergic signaling by mediating the release of ATP by the supporting cells [1]. How the disruption of these diverse functions contributes to the loss of hearing is still debated [14].

Most pathogenic variants resulting in DFNB1 NSHI lie within the coding region of *GJB2*. These include truncating variants (nonsense mutations, frameshifting small insertions and deletions) and non-truncating variants (missense mutations). A few pathogenic variants in splice sites or in the core promoter have also been reported. Finally, the spectrum of pathogenic variants also includes several large deletions, not involving *GJB2* but removing long DNA stretches far upstream of the gene (reviewed in [18]). These deletions are thought to remove a cis-acting regulatory element that would be essential for the expression of *GJB2* in the inner ear ([35, 42]). Deletion del(*GJB6*-D13S1830) was the first to be reported [16]. It encompasses 309 kb and eliminates the neighbouring genes *CRYL1*, encoding crystallin lambda 1, and *GJB6*, which codes for connexin-30 (Cx30), also a component of the gap junction networks of the inner ear. Deletion del(*GJB6*-D13S1830) has been found frequently in several populations, where it accounts for up to 8% of all DFNB1 pathogenic variants [17].

The degree of hearing loss in DFNB1 NSHI is widely variable. This is primarily a consequence of the type of causative mutation, as specific missense variants disrupt some but not all of the functions that Cx26 plays in the cochlea, as indicated above. But there must be other factors, as illustrated by homozygotes for the most common c.35delG mutation, whose hearing losses range from mild to profound [36]. It was hypothesized that this variability could be due to modifier genetic factors, but the identification of these modifiers remains elusive [25]. In spite of these difficulties, a broad genotype–phenotype correlation could be revealed, as truncating mutations produce more severe hearing losses than non-truncating mutations. Interestingly, the c.35delG/del(*GJB6*-D13S1830) genotype is consistently associated with a more severe (frequently profound) hearing loss [36].

Given the intricacies and epidemiological relevance of DFNB1 ARNSHI, obtaining a murine model to dissect in detail the DFNB1 pathological processes became an essential objective. The first attempt to analyse a *Gjb2* knockout (KO) mouse model generated by gene targeting quickly unraveled, because homozygous mutant mice died in utero at around day 11 post coitum [22]. Such embryonic lethality is due to the fact that murine Cx26, unlike its human equivalent, has an essential role in the transplacental transport of glucose, and maybe other nutrients, from maternal blood [22]. This difficulty was circumvented via conditional knockout (CKO) technology by the creation of a floxed model, *Gjb2*^*loxP/loxP*^ [15], that was crossed with mice which expressed Cre recombinase under the control of different promoters, driving *Gjb2* ablation in a cell type- or time-specific manner. Analysis of those models yielded valuable data on the pathological processes underlying Dfnb1 hearing impairment. However, there were significant phenotypic differences among models because it was not possible to obtain complete *Gjb2* ablation in all cochlear cell types synthesizing Cx26.

In spite of their epidemiological relevance and their unusual pathogenic mechanism, i.e. abolishing the expression of an otherwise intact *GJB2* gene in the inner ear, no murine model has been constructed to date for the far upstream large DFNB1 deletions. Here we report on the design, generation and characterization of the first Dfnb1 mouse model that imitates the del(*GJB6*-D13S1830) deletion. The hearing loss phenotype and the cochlear structural changes that we observed in homozygous mice are discussed in comparison with the findings from previous Dfnb1 models.

## Materials and methods

### Designing RNA guides and testing on Neuro2a murine cells

**Fig. 1:**
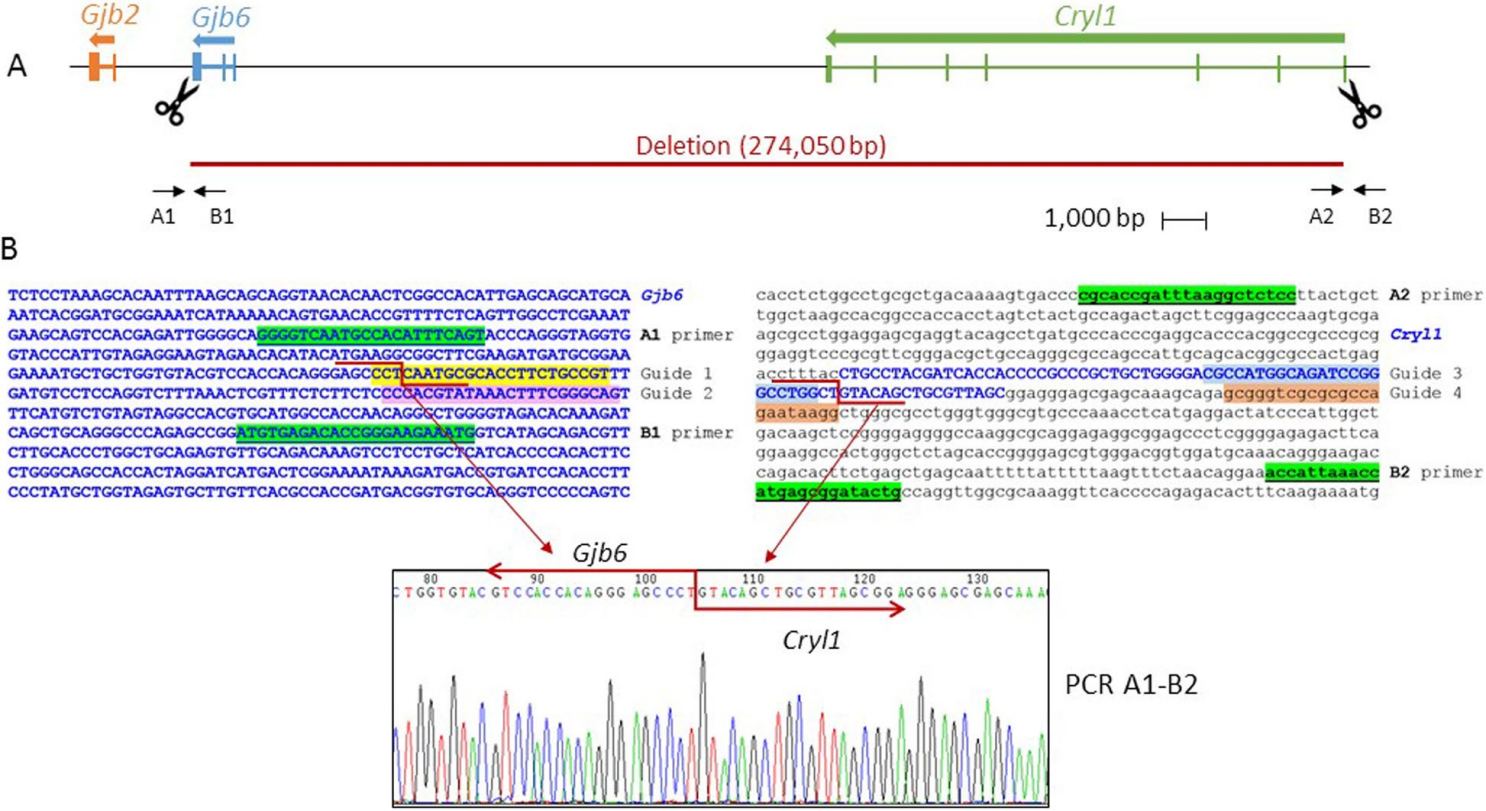
Deletion engineered at the murine *Dfnb1* locus. **A** Map of the chromosome 14 region targeted in this study, including genes of interest. Scissors indicate the localization of CRISPR guides, and arrows show the primers used to test for the deletion (primer arrows not to scale). **B** Genomic sequences of *Gjb6* exon 3 and *Cryl1* exon 1 regions, showing the location of the CRISPR guides, deletion breakpoints and primers that were used. Exonic sequences appear in blue bold capital letters, primers are highlighted in green background and underlined, and CRISPR guides are marked with different background colors. The actual breakpoints in each region are indicated by a red zigzag line, as deduced from the Sanger sequence of A1-B2 breakpoint PCR (bottom)

Selection of RNA guides for CRISPR experiments was performed by using the Breaking-Cas web tool [32], on the basis of having the highest arbitrary scores (> 95) and the fewest putative off-targets. To improve the efficiency of the edition, we used four guides (Table 1). Two guides were located in exon 3 of *Gjb6* and two others in exon 1 of *Cryl1* or just upstream, respectively (Fig. 1). The crRNAs were purchased from Integrated DNA Technologies (IDT, Coralville, IA, USA).

**Table 1:** Sequences and coordinates of the guides that were used for CRISPR edition

Guide	RNA sequence (5’-3’)	GRCm38/mm10 coordinates
G1 (*Gjb6*)	ACGGCAGAAGGUGCGCAUUG**AGG**	chr14:57,124,415–57,124,437
G2 (*Gjb6*)	CUGCCCGAAAGUUUAUACGU**GGG**	chr14:57,124,476–57,124,498
G3 (*Cryl1*)	CGCCAUGGCAGAUCCGGGCC**UGG**	chr14:57,398,443–57,398,465
G4 (*Cryl1*)	GCGGGUCGCGCGCCAGAAUA**AGG**	chr14:57,398,505–57,398,527

In bold, PAM sequences

A CRISPR assay was performed on murine neuroblastoma Neuro2a cells to evaluate the performance of the guides [23]. First, we verified by Sanger sequencing that the sequence of each of the four guide targets was indeed identical in the Neuro2a cell genome. Then, 200,000 cells were seeded per well in 4 wells of a P24 plate, each well containing 0.5 mL of complete medium (DMEM, supplemented with 10% fetal bovine serum, without antibiotics). Cells were incubated at 37 °C with 5% CO_2_ for 24 h. Cas9-RNP complexes were prepared in four tubes by adding (per tube): 500 ng of Cas9 protein (Alt-R S.p. HiFi Cas9 Nuclease V3, IDT, Coralville, IA, USA), 125 ng of Edit-R tracrRNA (Alt-R CRISPR-Cas9 tracrR, IDT, Coralville, IA, USA), 125 ng of each of the four crRNAs (Alt-R CRISPR-Cas9 crRNA, IDT, Coralville, IA, USA), 1 µl of P3000 reagent (Invitrogen, Waltham, MA, USA), and OptiMEM medium to a final volume of 25 µl. The mix was incubated for 5 min at room temperature. A lipofectamine solution (1.5 µl of Lipofectamine 3000 (Invitrogen, Waltham, MA, USA) in 23.5 µl of OptiMEM) was added to each tube of the Cas9-RNP complexes. After incubation for 15 min at room temperature, the final mix was added to the cells (50 µl per well). Cells were incubated at 37 °C with 5% CO_2_ for 3 days. DNA was extracted using the High Pure PCR Template Preparation Kit (Roche, Basel, Switzerland) following the manufacturer’s instructions.

To track the effects of the genomic editing three PCR assays were developed, using different combinations of four primers: A1 (5'-GGGGTCAATGCCACATTTCAGT-3'), B1 (5'-CATTTCTTCCCGGTGTCTCACAT-3'), A2 (5'-CGCACCGATTTAAGGCTCTCC-3') and B2 (5'-CAGTATCCGCTCATGGTTTAATGGT-3'). The A1-B1 assay amplified the region around the proximal breakpoint, whereas the A2-B2 assay amplified the region around the distal breakpoint. The A1-B2 combination allowed us to check whether the desired deletion had occurred (Fig. 1).

### CRISPR–Cas9 RNP microinjection into mouse fertilized eggs

A mix of 30 ng/µl of Cas9 mRNA (Dharmacon, Lafayette, CO, USA) and 15 ng/µl of each sgRNA (crRNA-tracrRNA) (Dharmacon, Lafayette, CO, USA) was prepared in sterile embryo-quality water. The final volume was adjusted to 50 μl using microinjection buffer (1 mM Tris–HCl pH 7.5; 0.1 mM EDTA pH 7.5), centrifuged for 30 min at 14,000 × g at 4ºC and kept on ice until use. Transgenic mice were produced by the CNB-CBMSO Transgenic Core Facility. RNA was microinjected into the cytoplasm of B6CBAF2 (Envigo; derived from intercrossing B6CBAF1/OlaHsd) fertilized oocytes [26] using standard procedures [2, 20, 23].

CRISPR reagents were microinjected into 72 mouse B6CBAF2 fertilized oocytes. Subsequently, the 27 surviving embryos (37.5%) were transferred into two foster females, resulting in the birth of 5 (18.5%) newborn mice.

### Mouse husbandry

All experimental procedures involving mice were validated by the local CNB-CSIC and IIBM-CSIC Ethics Committees on Animal Experimentation. These procedures were then favourably evaluated by the institutional CSIC Ethics Committee and approved by the Autonomous Government of Madrid, in accordance with Spanish (RD 53/2013) and European legislation. All mice were housed at the registered CNB animal facility, where they had ad libitum access to food (regular rodent chow) and water. They were maintained on a light/dark cycle of 08:00–20:00. Both male and female mice were used indistinctly in the experiments.

### Genotyping genome-edited mice

Mouse genomic DNA was extracted from tail biopsies (< 1 cm) using the High Pure PCR Template Preparation Kit (Roche, Basel, Switzerland) following the manufacturer's instructions. PCR assays to track the effects of the edition were performed as described for Neuro2a cells. All positive results were confirmed by sequencing. Selected positive mice were backcrossed to C57Bl/6JOlaHsd (Envigo, Indianapolis, IN, USA) for five consecutive generations. N5 mice were used to obtain homozygous mice for the intended deletions.

### Auditory function assessment

One month-old, male and female *Dfnb1*^*em274*^ homozygous (HOM, *n* = 6, three males and three females), heterozygous (HET, *n* = 12, nine males and three females) and wild type (WT, *n* = 10, nine males and one female) mice, in C57BL/6JOlaHsd (Envigo, Indianapolis, IN, USA) background, were included in the assessment. Control WT mice were littermates of the HOM mice. Auditory brainstem responses (ABR) recordings of one month-old mice were performed on a TDT ABR and DPOAE acquisition system, with a RZ6 processor (Tucker‐Davis Technologies, Alachua, FL, USA), as reported [5]. In brief, mice were anesthetized with ketamine (100 mg/kg; Imalgene 1000; Merial, Lyon, France) and xylazine (10 mg/kg; Rompun 2%; Bayer, Leverkusen, Germany) by intraperitoneal injection and the ABR tests were performed in a sound‐attenuating chamber. Two different sound stimuli, clicks and tone bursts, were generated with SigGenRZ software (TDT). Stimuli were calibrated using SigCalRZ software and a PCB 377C01 precision condenser microphone, with a 426B03 preamplifier and a 480C02 signal conditioner. Click (duration 0.1 ms) and tone burst (duration 5 ms, 2.5 ms each for rise and decay, without plateau) at 4, 8, 16, 24, 32 and 40 kHz stimuli were delivered by a MF1 open field magnetoelectrostatic speaker (TDT) at 30 (click) or 50 (tone bursts) pulses per second, and from 90 to 10 dB SPL, in 5–10 dB steps. The evoked response was collected with stainless steel needle electrodes placed at the vertex (active), ventrolateral to the right ear (reference) and tail base (ground), promediated, and analyzed with BioSigRZ software (TDT). ABR records for both click and pure tones were obtained after averaging 1000 evoked responses. Hearing thresholds were established at the lowest SPL level that produced a noticeable ABR five peaks wave and evoked a peak‐to‐peak voltage 2 SD above the mean background activity. When possible, wave amplitudes, latencies, and inter‐wave latencies were determined at 70 dB SPL click stimulation. Statistical analysis was performed with IBM SPSS statistics v29.0 software. Considering the sample size and the results of the Kolmogorov–Smirnov normality test, differences among genotypes were assessed by the nonparametric Kruskal–Wallis H test, and were considered significant at *p* < 0.05 (*p < 0.05, **p < 0.01, ***p < 0.01). We did not observe any differences between sexes. Analysis restricted to male animals (3 HOM, 9 HET, 9 WT) yielded the same results as those of males and females altogether.

### Cochlear morphology

For histological analysis, mice were anesthetized by intraperitoneal injection of pentobarbiturate (Dolethal, Vetoquinol, Spain) and then intracardially perfused with fresh phosphate-buffered saline (PBS) 0.1 M and pH 7.4, and 4% paraformaldehyde (PFA; Merck, Darmstadt, Germany) in PBS. Dissected inner ears were placed in fresh fixative solution (4% PFA) for 12 h, washed with PBS, decalcified in 5% EDTA (pH 6.5, Sigma‐Aldrich, St. Louis, MO, USA) for 7–10 days, and finally embedded in paraffin wax or resin (Historesin Standard kit, Leica #14,702,218,500) [7]. Paraffin cochlear midmodiolar serial Sects. (7 μm) were obtained on a RM2155 microtome (Leica Microsystems, Deerfield, IL, USA) and hematoxylin–eosin‐stained for gross cochlear cytoarchitecture. For details, historresin serial Sects. (2 μm) were obtained and stained with cresyl-violet. Paraffin sections were also used for immunodetection of connexin 26 (mouse anti-Cx26, invitrogen #13–8100, dilution 1:400) and connexin 30 (mouse anti-Cx30, invitrogen #70–0258, dilution 1:400), following the avidin–biotin-peroxidase (ABC) method using 3,3-DAB as chromogen [21]. Images were acquired with a Zeiss AxioPhot microscope (Carl Zeiss, Jena, Germany) and captured with an Olympus DP70 digital camera (Melville, NY, USA). Three mice of each genotype were assessed for historesin sections. Four mice of each genotype were assessed for paraffin sections. All of them were assessed at P30.

### Quantification of *Gjb2* expression in whole cochleae

The cochleae of the mice were frozen in RNAlater (Invitrogen, Waltham, MA, USA) for proper conservation, and processed for gene expression study as reported [6]. Briefly, total RNA was extracted from one cochlea from each mouse (two wild-type, two heterozygotes and two homozygotes). After quality control and fluorometer (QuBit) quantitation of the RNAs, cDNAs were synthesized with 300 ng total RNA as template and random hexamer primers, by using Superscript II reverse transcriptase (Life Technologies, Carlsbad, CA, USA) as recommended. Quantification of *Gjb2* expression was performed by real-time quantitative PCR (RT-qPCR) in a 7700 Real Time PCR System (Life Technologies, Carlsbad, CA, USA). Specific primer pairs were designed with Primer Express software v3.0 (Life Technologies, Carlsbad, CA, USA) for the following products: *Gjb2* (5’- CGCTCCTCCGGACACAGT-3’ and 5’- TGTTGACACCCCCGAGGAT-3’) and control *Hprt* (5’- GCAGTACAGCCCCAAAATGG-3’ and 5’- CAACACTTCGAGAGGTCCTTTTC-3’). For each assay, we analysed different amounts of cDNA (20, 100 and 500 ng) in duplicate. RT-qPCR reactions were carried out with 8 pmol of each primer by using the TB Green Premix Ex Taq (Tli RNase plus) qPCR kit (Takara Bio, Shiga, Japan) with the following amplification procedure: incubation at 50 °C (2 min); denaturation at 95 °C (10 min); 60 cycles of denaturation at 95 °C (15 s), annealing at 59 °C (30 s) and extension at 72 °C (30 s); and a final dissociation step of 95 °C (15 s), 60 °C (30 s) and 95 °C (15 s). Fluorescence was measured once per cycle, at the end of the extension step. Analysis of the relative amounts of *Gjb2* transcript as regards the expression of the reference gene *Hprt* was performed by the mathematical model of Pfaffl [34].

## Results

### Design of a targeted deletion at the *Dfnb1* locus and validation in murine Neuro2a cells

Our objective was to imitate as closely as possible the del(*GJB6*-D13S1830) deletion [16] (Fig. 1). We chose four guides, two on each side of the deletion, to be used simultaneously in the same assay, in order to improve the efficiency of the edition. The two proximal guides were selected on a 181-bp DNA segment within *Gjb6* exon 3, which contains the murine equivalent of the human proximal breakpoint, flanked by 90 bp on each side. To imitate the human distal breakpoint, guides could not be chosen on the exact murine homologous region, as it contains repeated elements that are undesirable targets for CRISPR guide selection. Instead, we designed guides on a closely-located region of unique sequence, which contains *Cryl1* exon 1 and a short upstream DNA stretch (Fig. 1).

The efficiency of the guides was tested by performing CRISPR-Cas9 mutagenesis on murine Neuro2a cells. The occurrence of the desired deletion was tracked through PCR amplification, as described in Materials and Methods. The A1-B2 products, only amplifiable if the deletion had occurred (Fig. 1), were obtained and sequenced, confirming that deletions had taken place between the expected breakpoints. Therefore, the guides were appropriate to generate a *Dfnb1* deletion allele in mice.

### Generation of mice harbouring the *Dfnb1*^*em274*^ allele

A mix of combined CRISPR reagents (Cas9 mRNA and each of the four sgRNAs) was injected into 72 mouse B6CBAF2 fertilized oocytes. 27 surviving embryos were transferred into two foster mothers, which eventually gave birth to 5 founder mice (18.5%).

We extracted genomic DNA from tail biopsies and genotyped the progeny of the 5 founder mice with the three PCR assays that are described in Materials and Methods (Fig. 1; Suppl. Figure 1). Only one of the five founders, male B9690, was able to transmit the deleted allele to the progeny. Sequencing of the A1/B2 PCR product identified with precision the deletion breakpoints (Fig. 1B), revealing a deletion that spans 274,050 bp from *Gjb6* exon 3 to *Cryl1* exon 1. We termed this allele *Dfnb1*^*em274*^.

Heterozygous *Dfnb1*^*em274*^ mice were backcrossed to C57BL/6JOlaHsd for five consecutive generations. Upon reaching N5 (which actually corresponds to a minimum of 98,4% C57BL/6J purity), heterozygous mice were subsequently intercrossed to generate *Dfnb1*^*em274*^ homozygotes (HOM), which were born at the expected Mendelian frequency. Indeed, *Dfnb1*^*em274*^ HOM were viable, fertile and otherwise indistinguishable from their wild-type (WT) or heterozygous (HET) littermates.

### Hearing loss in *Dfnb1*^*em274*^ homozygotes

**Fig. 2:**
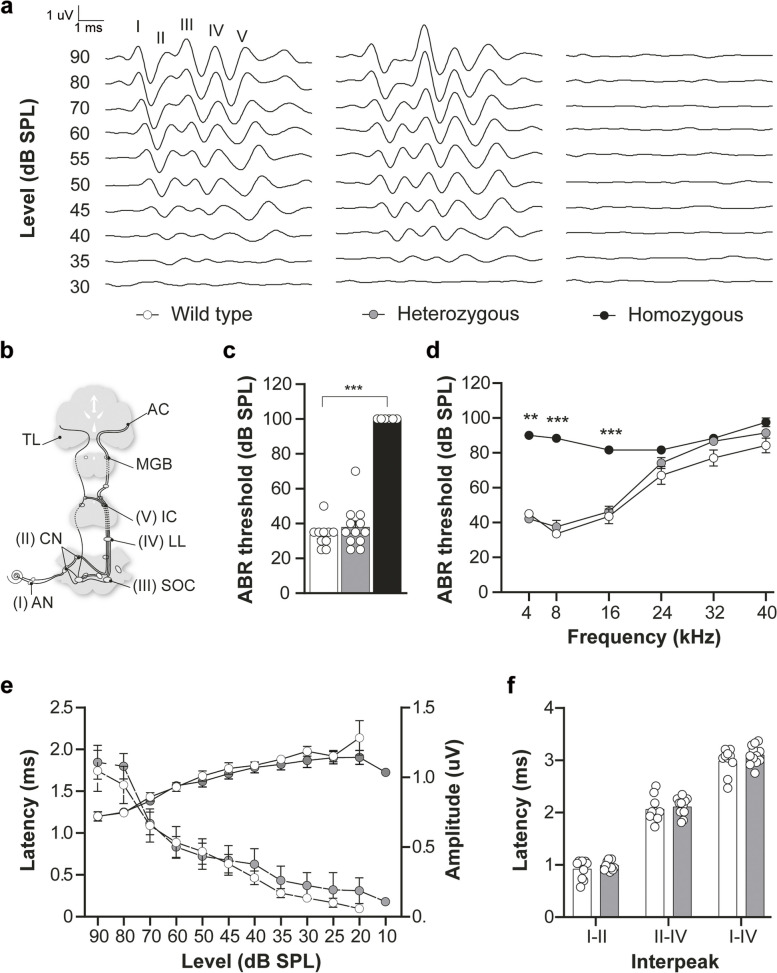
Auditory phenotype of P30 *Dfnb1*^*em274*^ HOM mice. **a** ABR in response to click stimulus at different level from 90 to 30 dB SPL, in WT, HET and HOM *Dfnb1*^*em274*^ mice. Latin numbers (I-V) indicate the standard ABR peaks. **b** Scheme showing the main neural centers in the auditory pathway and their correspondence with the ABR wave peaks (AN (I): auditory nerve; CN (II), cochlear nucleus; SOC (III): superior olivary complex; LL (IV): lateral lemniscus; IC (V), inferior colliculus; NLL: Lateral lemniscus nuclei; MGB, medial geniculate body; SC, superior colliculus; TL, temporal lobe; AC, auditory cortex. **c**, **d** ABR thresholds (in dB SPL) in response to click (**c**) and 4–40 kHz pure tones (**d**) in WT (white), HET (grey) and HOM (black) *Dfnb1*^*em274*^ mice. Statistically significant differences in click and 4, 8 and 16 kHz thresholds were found between WT and HOM mutant mice. (**e**) Input–output curves (latency/intensity and amplitude/intensity) for ABR peak I and (**f**) wave interpeak latencies I-II, II-IV and I-IV in WT and HET mutant mice

We next evaluated hearing in P30 *Dfnb1*^*em274*^ mutant HOM, HET and WT mice. *Dfnb1*^*em274*^ HOM mice showed a severe to profound hearing loss, with significantly increased ABR thresholds in response to click and tone-burst stimuli, compared to age-matched normal-hearing WT mice (Fig. 2a-c). No ABR responses were obtained after click stimulation, even at the maximum sound intensity employed (90 dB SPL), in any of the HOM mice included in the study, thus a generic 100 dB SPL value was annotated for statistical analysis. ABR thresholds for 4–16 kHz (Fig. 2d) were also significantly elevated compared to wild-type (*p* < 0.001). *Dfnb1*^*em274*^ HET mice presented a hearing phenotype similar to WT, with normal hearing thresholds (< 45 dB SPL) for click and low frequency (4–16 kHz) stimuli, and elevated thresholds for high frequency (24–40 kHz) tones. No significant differences in ABR thresholds were found between HET and WT mice.

We calculated the latency and amplitude of ABR waves I to V, which correspond to different nuclei in the auditory pathway in the brainstem (Fig. 2b), in the ABR recordings obtained in response to click stimuli, from 90 to 10 dB SPL in HET and WT mice. We plotted latency/intensity and amplitude/intensity curves for ABR wave I (Fig. 2e) and found no differences between HET and WT genotypes, with the expected physiological increase of the latency and decrease of the amplitude with the click level. Similarly, we calculated interpeak latencies between waves I-II, II-IV and I-IV in response to click stimuli at 70 dB, again without any differences between HET and WT mice (Fig. 2f).

### *Dfnb1*^*em274*^ homozygotes present gross abnormalities in cochlear morphology

**Fig. 3:**
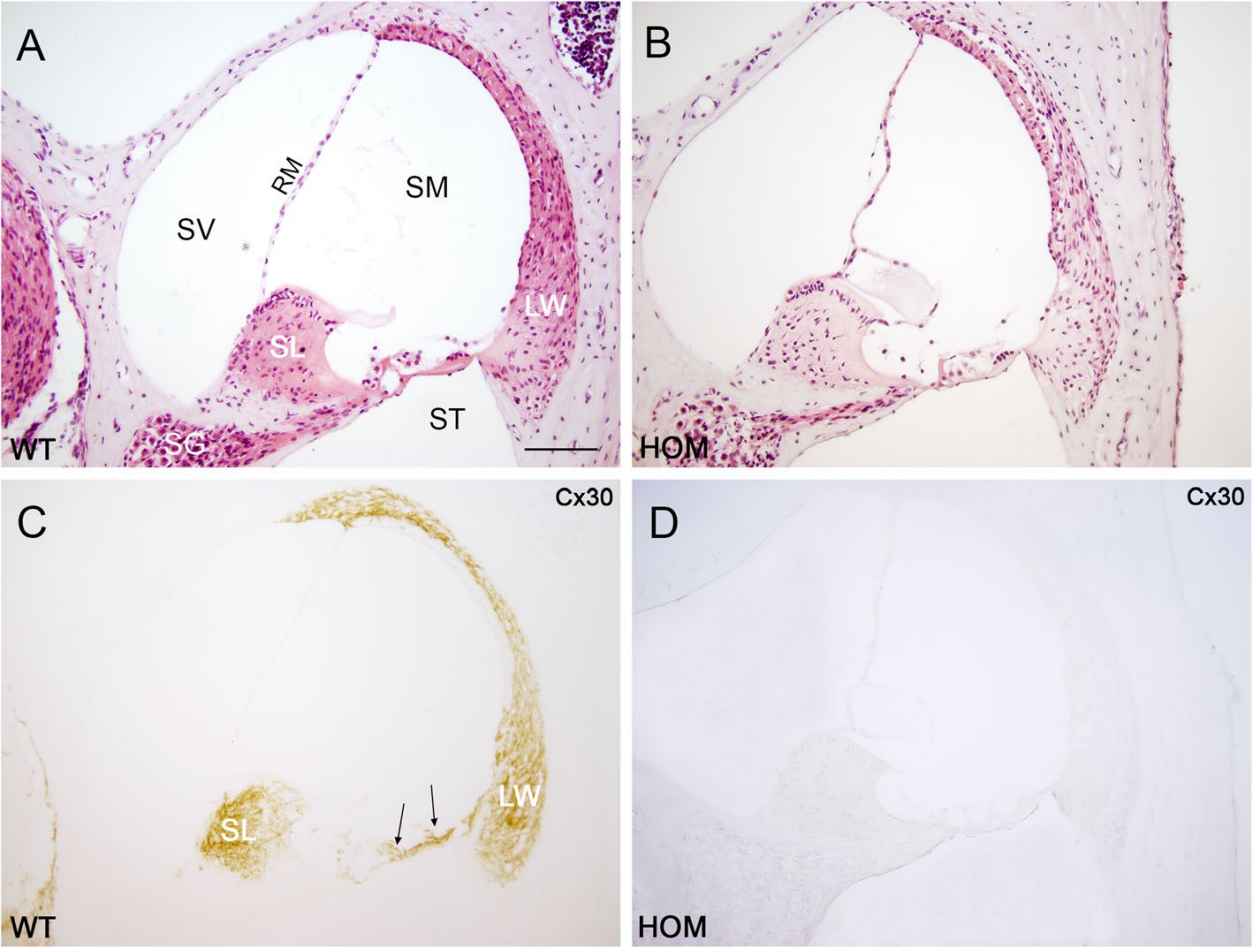
Cx30 expression in the cochlea of P30 *Dfnb1*^*em274*^ HOM mice. Representative micrographs of cochlear paraffin sections stained with hematoxylin–eosin (**A**, **B**) or immunostained against Cx30 (**C**, **D**), showing the basal region of WT (**A**, **C**) and *Dfnb1*^*em274*^ HOM (**B**, **D**) mice. WT mice present a normal cochlear cytoarchitecture and Cx30 expression in the spiral limbus, organ of Corti (surrounding supporting cells, arrows in **C**) and lateral wall. *Dfnb1*^*em274*^ HOM mice show a complete absence of Cx30 expression (**D**) and gross alterations in the Reissner membrane, tectorial membrane, and organ of Corti, compared to WT (**B**). LW, lateral wall; RM, Reissner membrane; SG, spiral ganglion; SL, spiral limbus; SM, scala media; ST, scala tympani; sTV, stria vascularis; SV, scala vestibuli. Scale bar: 100 mm

**Fig. 4:**
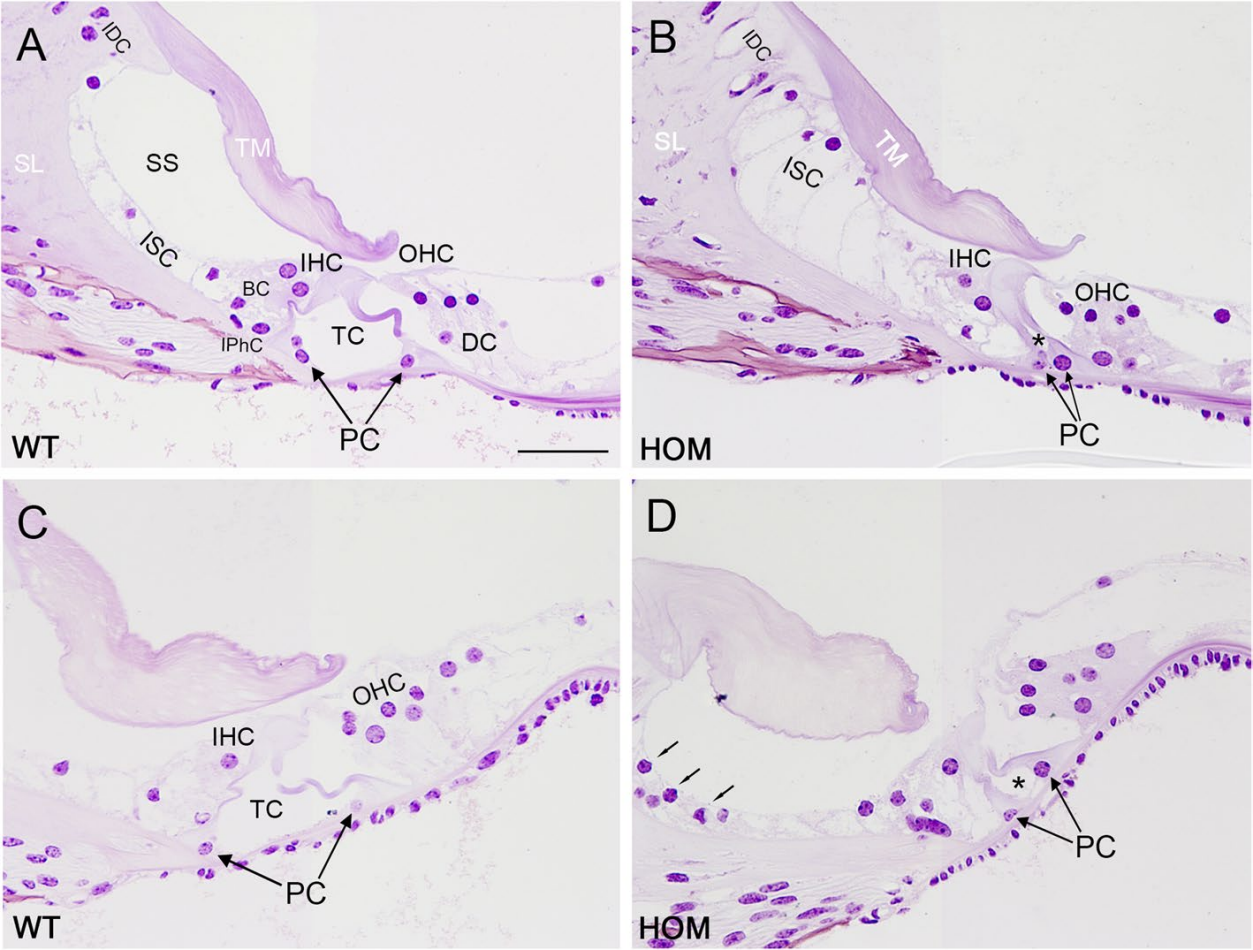
Organ of Corti cytoarchitecture in P30 *Dfnb1*^*em274*^ HOM mice. Representative micrographs of historesin cochlear sections stained with cresyl violet, showing the organ of Corti at the basal (**A**, **B**) and apical (**C**, **D**) regions in WT (**A**, **C**) and *Dfnb1*^*em274*^ HOM (**B**, **D**) mice. WT mice present normal Corti cytoarchitecture, whereas *Dfnb1*^*em274*^ HOM mice show an absent spiral sulcus (SS) with presence of large inner sulcus cells, vacuolated interdental cells, and reduced or collapsed tunnel of Corti (TC, asterisks in **B**, **D**) due to the lack of separation between pillar cells (PC, arrows in **B**, **D**). These alterations are most severe in the basal turn of the cochlea. At apex region, the homozygous mouse shows spiral sulcus separated from the tectorial membrane, inner sulcus cells lower than in more basal regions (small arrows in **D**), and small tunnel of Corti (asterisk in **D**) with separate pillars. BC, border cells; DC, Deiter cells; IDC, interdental cells; IHC, inner hair cells; IPhC, inner phalangeal cells; ISC, inner sulcus cells; OHC, outer hair cell; PC, pillar cells; SL, spiral limbus; SS, spiral sulcus (inner sulcus); TC, tunnel of Corti; TM, tectorial membrane. Scale bar: 50 mm

We analyzed cochlear gross morphology of P30 *Dfnb1*^*em274*^ HOM, HET and WT mice in resin and paraffin sections stained with cresyl violet and hematoxylin–eosin respectively. Compared to WT mice, *Dfnb1*^*em274*^ HOM mice presented major cytoarchitecture aberrations in their cochleae (Fig. 3A, B), especially at the organ of Corti and nearby regions, including: a thickened tectorial membrane; large inner sulcus cells, attached to the tectorial membrane; absence or reduction of the internal spiral sulcus; and joined internal and external pillars and absence or reduction of tunnel of Corti, compared to WT (Fig. 4). These findings were more evident in the basal and middle cochlear turns, and appear to a lesser extent in the apical turn. Inner and outer hair cells, as well as their supporting cells, appear normal, although some outer hair cells are lost in certain regions (Suppl. Figs. S2 & S3). Most of the HOM mice also showed a larger Reissner membrane, sometimes folded (Fig. 3B). Morphological alterations in HOM mice are reminiscent of immature cochlear structures observed during early postnatal development in mouse, suggesting a lack of maturation of key elements for normal cochlear physiology in *Dfnb1*^*em274*^ HOM mice. In general, *Dfnb1*^*em274*^ HET mice showed normal cochlear structure and size that were similar to those of the WT, with no evident malformations in the scala media.

### Cochlear Cx26 levels and *Gjb2* expression are drastically reduced in *Dfnb1*^*em274*^ homozygotes

**Fig. 5:**
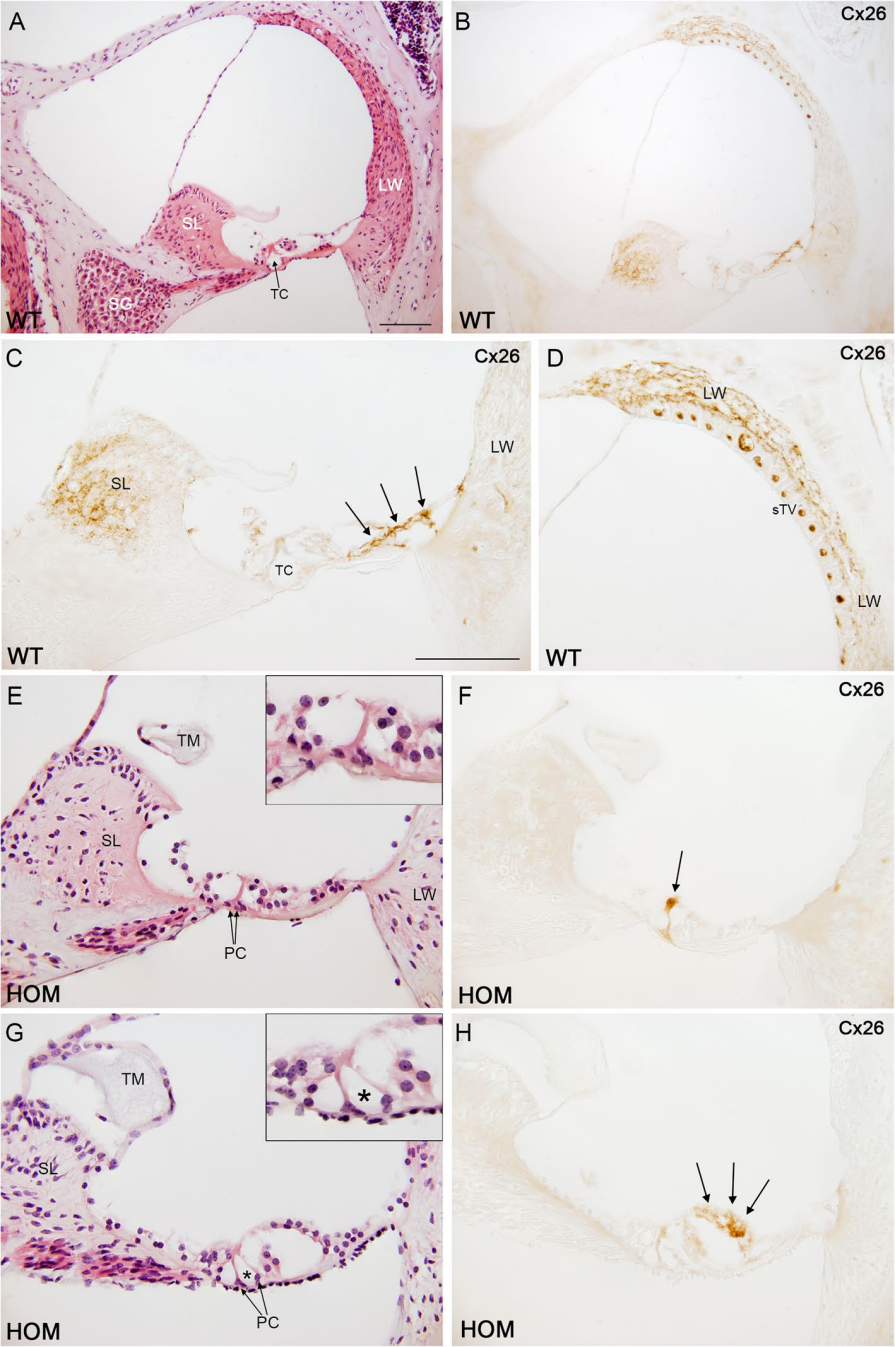
Cx26 expression in the cochlea of P30 *Dfnb1*^*em274*^ HOM mice. Representative micrographs of cochlear paraffin sections stained with hematoxylin–eosin or immunostained against connexin-26 in WT (**A-D**) and *Dfnb1*^*em274*^ HOM mice (**E–H**). WT mice show normal cochlear cytoarchitecture (**A**) and connexin 26 expression (**B**) in the spiral limbus (**C**), organ of Corti (supporting cells, arrows in **C**) and lateral wall (**D**), both in basal (**A-D**) and apical (not shown) regions. In contrast, *Dfnb1*.^*em274*^ HOM mice present gross morphological alterations such as collapse of the tunnel of Corti (insets in **E** and **G**, asterisk in **G**) and a drastic reduction in connexin-26 expression, which is detected residually in just a few pillar cells (arrow in **F**) and supporting cells (arrows in** H**). These changes were most severe in the basal turn (**E–F**) compared to apical turn (**G-H**). LW, lateral wall; PC, pillar cells; SG, spiral ganglion; SL, spiral limbus; sTV, stria vascularis; TC, tunnel of Corti; TM, tectorial membrane. Scale bar: 100 μm (**A-B**) and 50 μm (**C-H**)

**Fig. 6:**
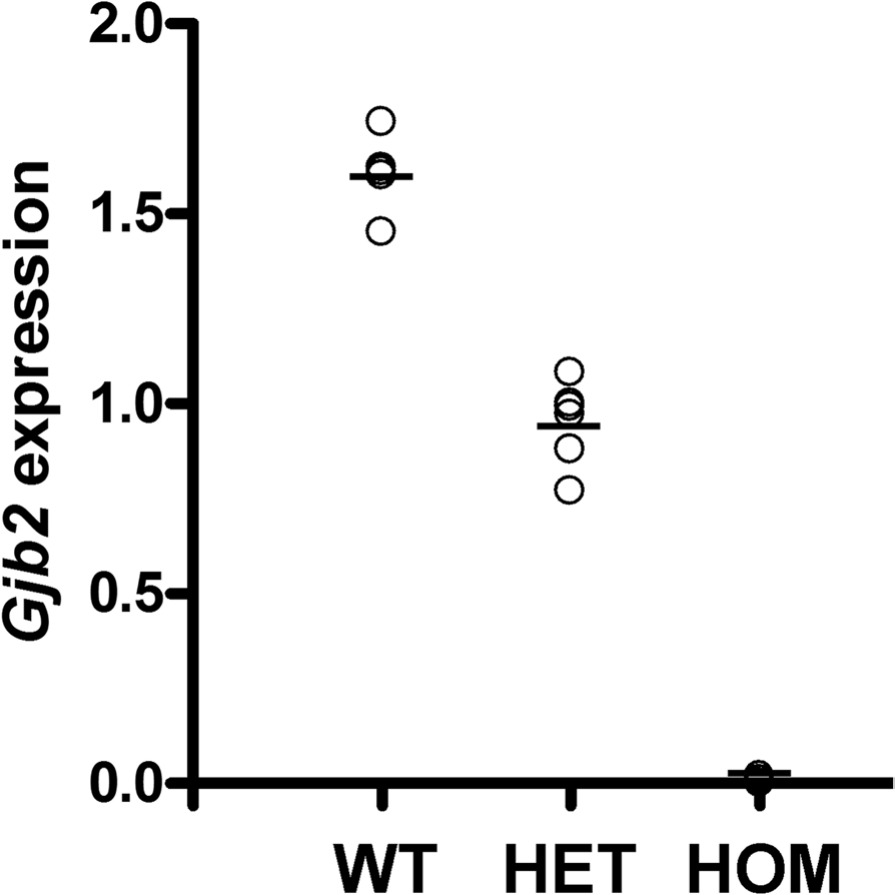
*Gjb2* expression in the whole cochleae of wild-type (WT), heterozygous (HET), and homozygous (HOM) P30 *Dfnb1*^*em274*^ mice. *Gjb2* expression was quantified by using real-time qPCR. The Y axis indicates the relative amounts of *Gjb2* transcript with regard to the expression of the reference gene *Hprt*, as calculated according to the mathematical model of Pfaffl [34]. Points on the graph correspond to three different cDNA concentrations for one cochlea from each mouse (2 WT, 2 HET, 2 HOM), each point being the mean of two independent experiments. Horizontal lines show the mean of the expression level of *Gjb2* for each genotype

We carried out immunodetection for connexins 30 and 26 in paraffin sections. Cx30 immunostaining in WT mice was located mostly in the spiral limbus, lateral wall and organ of Corti (Fig. 3C and Suppl. Fig. S4). Cx30 was completely absent in *Dfnb1*^*em274*^ HOM mice (Fig. 3D), as expected. As for Cx26, immunostaining in the WT was located in the same cochlear regions than Cx30 (spiral limbus, organ of Corti and lateral wall), although to a lesser extent (Fig. 5A-D and Suppl. Fig. S5). Interestingly, *Dfnb1*^*em274*^ HOM mice showed a drastic reduction in the staining of Cx26, which was almost absent in the organ of Corti, spiral limbus and lateral wall of the HOM mice cochlea (Fig. 5E-H). The reduction in Cx26 staining in *Dfnb1*^*em274*^ HOM mice was more evident in the basal cochlear turn, where the alterations in the tunnel of Corti were most severe (Fig. 5F & H). To ascertain whether this reduction in Cx26 levels happened at the transcriptional level, we investigated *Gjb2* expression in WT, HET and HOM *Dfnb1*^*em274*^ mice (two each) by real-time quantitative PCR (RT-qPCR) performed on cDNA synthesized from RNA extracted from whole cochleae (Fig. 6). We observed that *Gjb2* expression was nearly abolished in *Dfnb1*^*em274*^ HOM mice and that HET mice showed a reduction in *Gjb2* transcription to about half of mean WT level.

## Discussion

In this work we have successfully generated a mouse model imitating del(*GJB6-*D13S1830), the most frequent of the large deletions that result in DFNB1 ARNSHI. Heterozygous *Dfnb1*^*em274*^ mice do not differ from their wild-type littermates in auditory thresholds and inner ear cytoarchitecture, showing that the *Dfnb1*^*em274*^ allele is recessive, like its human counterpart. The auditory phenotype of homozygous *Dfnb1*^*em274*^ mice is essentially identical to that observed in del(*GJB6-*D13S1830) homozygous or c.35delG/del(*GJB6-*D13S1830) compound heterozygous human subjects, i.e. profound hearing loss across all frequencies.

Early attempts to generate a *Gjb2* knockout (KO) mouse model by gene targeting were hampered by the concomitant disruption of the transplacental transport of glucose that led to embryonic lethality [22]. This obstacle was circumvented through the generation of conditional knockout (CKO) models based on the floxed *Gjb2*^*loxP/loxP*^ mouse [15] by excising *Gjb2* just in specific cochlear cell types - with promoters such as *Otog*-Cre [15], *Foxg1*-Cre or *Pax2*-Cre [41] – or after birth, with tamoxifen-inducible promoters for Cre expression [10–13, 37, 43]. The two CKO approaches, though, could not guarantee *Gjb2* ablation in all cochlear cell types synthesizing Cx26, resulting in phenotypic differences among CKO models (Table 2). In addition to these KOs, a knock-in (KI) model was constructed to investigate the effects of the highly frequent c.35delG truncating *GJB2* mutation (Table 2). This KI mouse was made viable by performing enhanced tetraploid embryo complementation, a complex process with low success rate (just 1.78% of reconstituted embryos survived into adulthood) [28]. In contrast, our *Dfnb1*^*em274*^ model keeps the structure of *Gjb2* intact, but abolishes its expression almost completely, leading to a nearly absent Cx26 staining in the organ of Corti, spiral limbus and lateral wall (including stria vascularis) in HOM mice. In fact, the deletion was hypothesized to remove cis-acting regulatory sequences that are essential for Cx26 expression at least in the inner ear [31, 35, 42]. The *Dfnb1*^*em274*^ mice are viable and fertile, suggesting that the embryonic lethality does not occur because either those regulatory sequences would be essential for *Gjb2* expression only in the cochlea or they would be dispensable in the placenta. In any case, our model successfully targets both the epithelial and the connective-tissue gap junction networks in the cochlea, in contrast to CKO models, as indicated above.

**Table 2:** Characteristics of DFNB1 mouse models

Model genotype	*Gjb2*^*loxP/loxP*^ *Otog*-Cre	*Gjb2*^*loxP/loxP*^ *Foxg1*-Cre or *Pax2*-Cre	*Gjb2*^*loxP/loxP*^ *Rosa26*^*CreERT*^	*Gjb2*^*loxP/loxP*^ *ROSA26*^*Cre/Esr1*^	*Gjb2* c.35delG KI	*Dfnb1*^*em274/em274*^
Type	CKO	CKO	TMX-inducible CKO	TMX-inducible CKO	KI	KO
Cochlear structures without Cx26	Organ of Corti	Organ of Corti & spiral limbus	Organ of Corti, KD elsewhere	Organ of Corti & spiral limbus	All	All
Cx30 expression	Normal	Normal	Normal	Normal	Reduced	Abolished
Hearing loss (ABR threshold in dB SPL)	Severe (70), all frequencies	Severe (80), all frequencies	Severe (80), all frequencies	Profound (> 110), all frequencies	Profound (> 80), all frequencies	Profound (> 90), all frequencies
Age at ABR	P30	P21 & P84	P21, P30, P60, P120	P14, P20, P30	P14 & P35	P30
Organ of Corti	Degeneration with missing supporting cells	Degeneration with missing supporting cells, no TC	Degeneration with missing supporting cells	Degeneration with missing supporting cells, no TC	Immature, no TC	Immature, no TC
Hair cells	Death of OHC, intact IHC	Death of OHC, intact IHC	Death of OHC, intact IHC	Not reported	Some OHC loss, intact IHC	Some OHC loss, intact IHC
References	Cohen-Salmon et al. 2002 [15]	Wang et al. 2009 [41] Chang et al. 2015 [10] Liang et al. 29	Sun et al. 2009 [37] Chen et al. 2014a [11] Chang et al. 2015 [10] Chen et al. 2018 [13]	Chen et al. 2014b [12] Zhu et al. 2015 [43]	Li et al. 2023 [28]	**This work**

*Abbreviations*: *CKO* Conditional knockout, *KI* knockin, *KO* knockout, *TMX* tamoxifen, *KD* knockdown, *TC* tunnel of Corti, *OHC* outer hair cells, *IHC* inner hair cells

Of note, our model does remove the neighbouring *Gjb6* gene, whose product, Cx30, also participates in the gap junction networks of the inner ear. However, it is known that Cx30 is dispensable for hearing in mice [4] and that the severe hearing loss observed in a prior *Gjb6* knockout model [40] was caused by a polar effect of the engineered mutation on the *Gjb2* gene located downstream, provoking a strong reduction in the synthesis of *Gjb2* transcripts and of Cx26 [4]. Nevertheless, it is possible that Cx30 may play some role in attenuating the effects of the absence of Cx26, and so the lack of both connexins may be responsible for the more severe phenotype (profound deafness) that is associated with the deletion.

CKO models based on the *Gjb2*^*loxP/loxP*^ mouse show a degeneration of the organ of Corti with missing supporting cells, the death of outer hair cells (OHC) and surviving inner hair cells (IHC), plus a concomitant severe hearing loss (ABR thresholds at 70–80 dB SPL) across all frequencies (Table 2). Although the loss of the endocochlear potential due to the disruption of the epithelial barrier in the organ of Corti was originally suggested as one of the causes for this hearing loss, subsequent work indicated instead that the major cause was a lack of maturation of the synaptic machinery of IHC [10, 12, 15, 29]. In contrast, the *Dfnb1*^*em274*^ model shows a markedly different phenotype in which the final structural development of the organ of Corti is arrested, the tunnel of Corti does not open, supporting cells do not degenerate and all IHC and most OHC survive. In the CKO models, Cx26 is absent just in the organ of Corti (*Otog*-Cre) or in the organ of Corti and the spiral limbus (*Foxg1*-Cre, *Pax2*-Cre, *Rosa26*^*CreERT*^, *Rosa26*^*Cre/Esr1*^) and Cx30 expression throughout the cochlea remains normal. In the *Dfnb1*^*em274*^ model, though, Cx26 is absent from nearly all cochlear structures, and *Gjb6* is knocked out. Indeed, all five known roles for connexins 26 and 30 within the cochlea are effectively eliminated in *Dfnb1*^*em274*^ mice, which results in a structurally and functionally immature cochlea and the profound deafness phenotype observed (ABR thresholds > 90 dB SPL). The phenotype of the c.35delG KI is similar to that of the *Dfnb1*^*em274*^ mouse, with an immature sensory epithelium without tunnel of Corti, surviving IHC, mostly intact OHC and profound hearing loss (slightly lower ABR thresholds at 80 dB SPL) across all frequencies. Intriguingly, cochlear Cx30 expression in the c.35delG KI was reported as downregulated all throughout the cochlea from P14 onward [28], a co-regulation between Cx26 and Cx30 first described by Ortolano et al. [33]. It is tempting to speculate that the difference in the severity of the hearing loss between c.35delG KI and *Dfnb1*^*em274*^ models, albeit small (ABR thresholds at 80 dB SPL versus > 90 dB SPL), is due to differences in the cochlear levels of Cx30 (downregulated versus abolished). If it were true, the expression levels of Cx30 may be a modifier factor that could contribute to the phenotypic variability that is observed among subjects carrying the same *GJB2* genotype.

In conclusion, the *Dfnb1*^*em274*^ mouse faithfully recapitulates the effects of the prevalent, loss-of-function del(*GJB6-*D13S1830) human variant, providing a straightforward and easy-to-breed model to investigate mechanisms of pathogenicity and assay possible therapies for DFNB1 ARNSHI. Indeed, the del(*GJB6-*D13S1830) allele keeps structurally intact the *GJB2* gene, and so it could be amenable to strategies intended to reactivate its expression.

### Supplementary Information

**Supplementary Material 1. **

## Data Availability

B6.CBA-Dfnb1em274/Lmon CRISPR genome-edited mice are available directly from Lluis Montoliu’s laboratory at CNB-CSIC in Madrid (Spain) and will be deposited shortly in the European Mouse Mutant Archive (EMMA-Infrafrontier; https://infrafrontier.eu/) from where these mice will be made available to the entire scientific community. Mouse Genome Informatics (MGI) database accession number: MGI:7,581,999 (synonym Del(14Gjb6-Cryl1)1Lmon).
