# Supplementary material for: A murine model for the del(GJB6-D13S1830) deletion recapitulating the phenotype of human DFNB1 hearing impairment: generation and functional and histopathological study
Source: BMC Genomics. 2024 Apr 11;25:359. doi: 10.1186/s12864-024-10289-z (PMC11007912; doi:10.1186/s12864-024-10289-z)
Supplement: Supplementary file 1 — Supplementary Material 1. [file 12864_2024_10289_MOESM1_ESM.docx]

**SUPPLEMENTARY FIGURES**

María Domínguez-Ruiz^1,6,†^, Silvia Murillo-Cuesta^2,3,6,†^, Julio Contreras^2,4,6,†^, Marta Cantero^5,6^, Gema Garrido^1,5,6^, Belén Martín-Bernardo^2,3,6^, Elena Gómez-Rosas^1^, Almudena Fernández^5,6^, Francisco J. del Castillo^1,6^, Lluís Montoliu^5,6^, Isabel Varela-Nieto^2,3,6^, Ignacio del Castillo^1,6, *^

^1^ Servicio de Genética, Hospital Universitario Ramón y Cajal, IRYCIS, Madrid, Spain.

^2^ Institute for Biomedical Research "Sols-Morreale", Spanish National Research Council-Autonomous University of Madrid, Madrid, Spain.

^3^ Hospital La Paz Institute for Health Research (IdiPAZ), Madrid, Spain

^4^ Anatomy and Embryology Department, Faculty of Veterinary, Universidad Complutense de Madrid, Madrid, Spain.

^5^ Department of Molecular and Cellular Biology, National Centre for Biotechnology (CNB-CSIC), Madrid, Spain.

^6^ Centro de Investigación Biomédica en Red de Enfermedades Raras (CIBERER-ISCIII), Madrid, Spain.

† These three authors contributed equally to this work and must be considered first co-authors

^*^ Corresponding author: Dr. Ignacio del Castillo ([ignacio.castillo@salud.madrid.org](mailto:ignacio.castillo@salud.madrid.org))


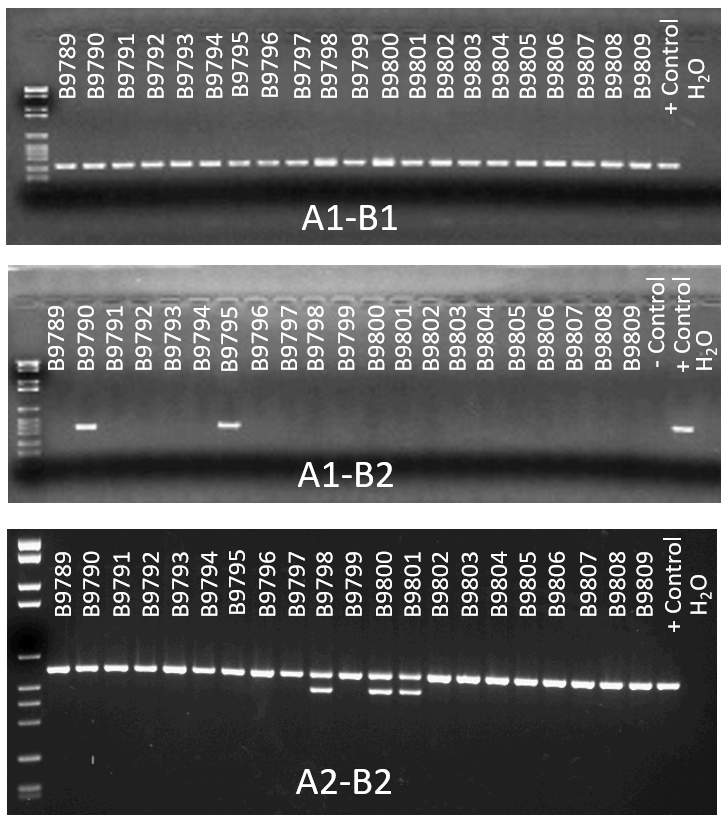


**Suppl. Fig. S1**. **Representative example of genotyping PCRs of N1 generation mice.** PCR amplification with A1/B1 and A2/B2 primer pairs indicates the presence of at least one intact (undeleted) allele. In PCR A2/B2, the smaller bands that appear in individuals B9798, B9800 and B9801 are due to very small deletions that occurred between the two guides at the distal (*Cryl1*) breakpoint site. PCR amplification with the A1/B2 primer pairs indicates that the individual carries the desired allele with the deletion. Altogether, the results of the three PCR experiments show that mice B9790 and B9795 are heterozygous for the desired deletion as the expected amplicons are obtained with all three A1/B2, A1/B1 and A2/B2 primer pairs


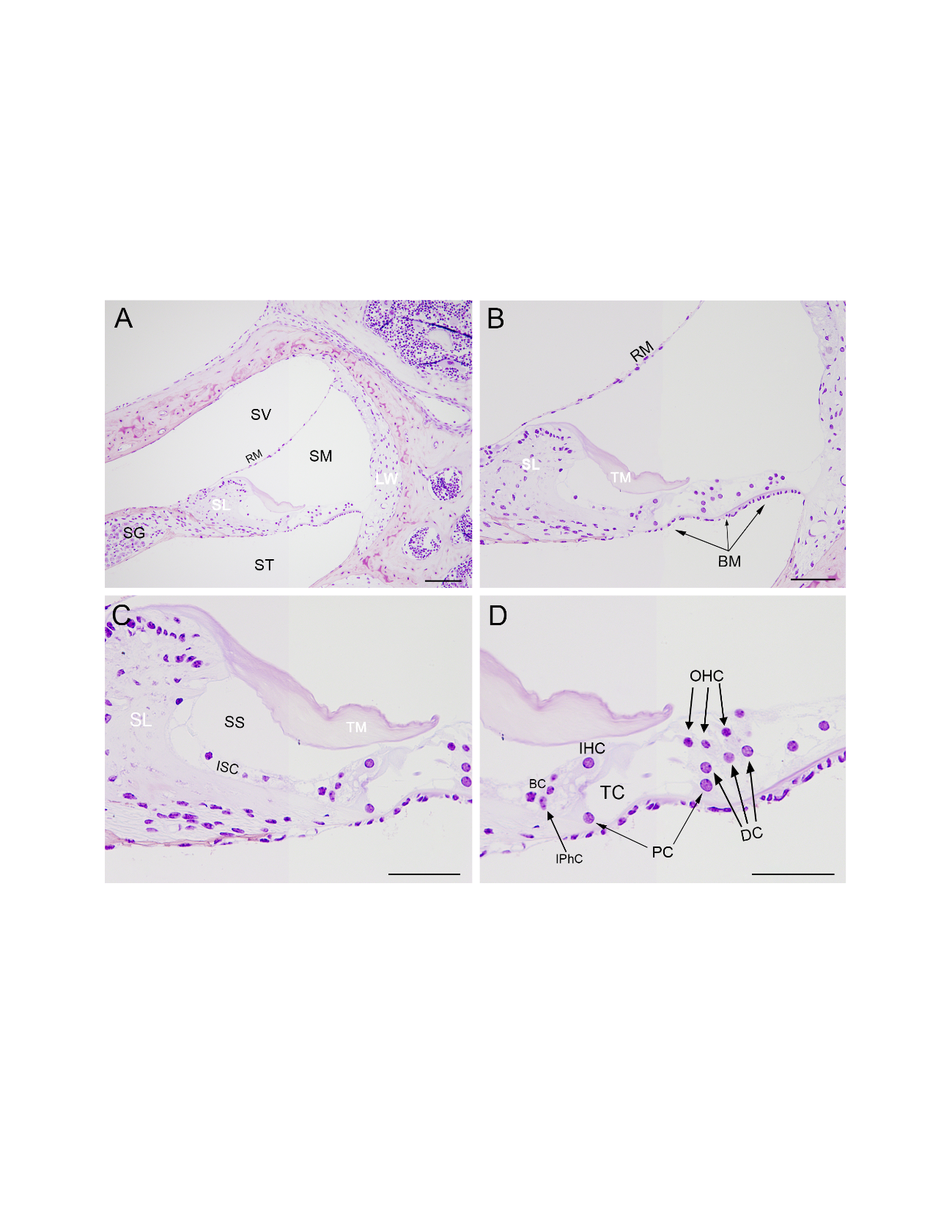


**Suppl. Fig. S2. Cochlear morphology in P30 WT mice.** Micrographs of historesin cochlear sections stained with cresyl violet, showing the basal/middle cochlear turn (**A**) and magnification of the scala media (**B**), which is separated from the scala vestibuli by the Reissner membrane and from the scala tympani by the basilar membrane. Magnification of the spiral limbus and spiral sulcus region (**C**), with normal cytoarchitecture of the inner sulcus cells and interdental cells. (**D**) Detail of the organ of Corti with sensory hair cells and accompanying supporting cells. Pillar cells sustaining the tunnel of Corti are also visible. BC, border cells; BM, basilar membrane; CC, Claudius cells; DC, Deiter cells; HC, Hensen cells; IDC, interdental cells; IHC, inner hair cells; IPhC, inner phalangeal cells; ISC, inner sulcus cells; OHC, outer hair cell; PC, pillar cells; RM, Reissner membrane; SG, spiral ganglion; SL, spiral limbus; SM, scala media; SS, spiral sulcus (inner sulcus); ST, scala tympani; SV, scala vestibuli; TC, tunnel of Corti; TM, tectorial membrane. Scale bar: 100 μm (A) and 50 μm (B-D)


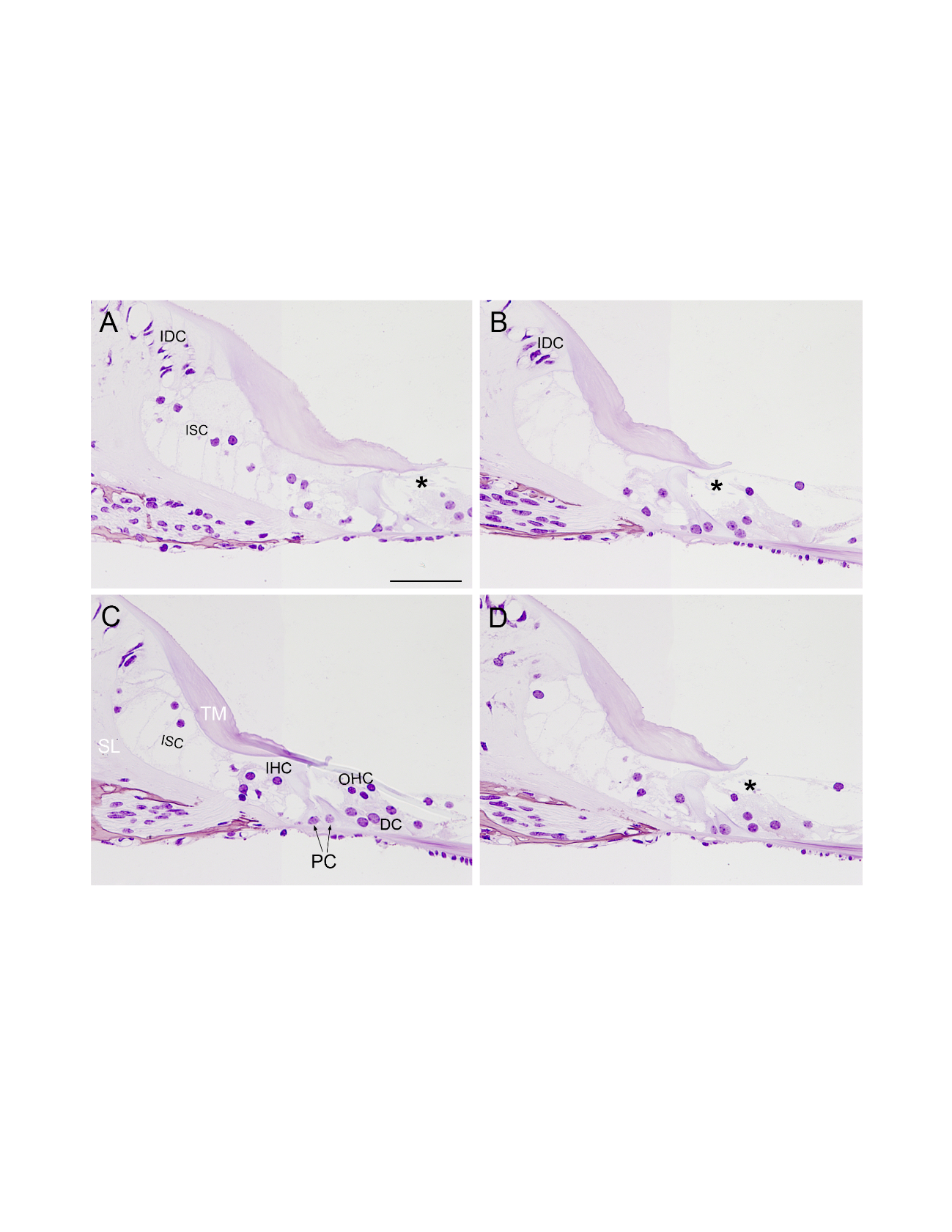


**Suppl. Fig. S3. Cochlear morphology in P30 *Dfnb^em274^* HOM mice.** Micrographs of historesin sections stained with cresyl violet at the basal/middle cochlear turn, showing in detail the aberrant morphology of inner sulcus cells, the collapse of the tunnel of Corti and the presence of immature interdental cells, with large vacuoles and elongated nuclei. In some animals, loss of outer hair cells is observed (asterisk). DC, Deiter cells; IDC, interdental cells; IHC, inner hair cells; ISC, inner sulcus cells; OHC, outer hair cell; PC, pillar cells; SL, spiral limbus; TM, tectorial membrane. Scale bar: 50 μm


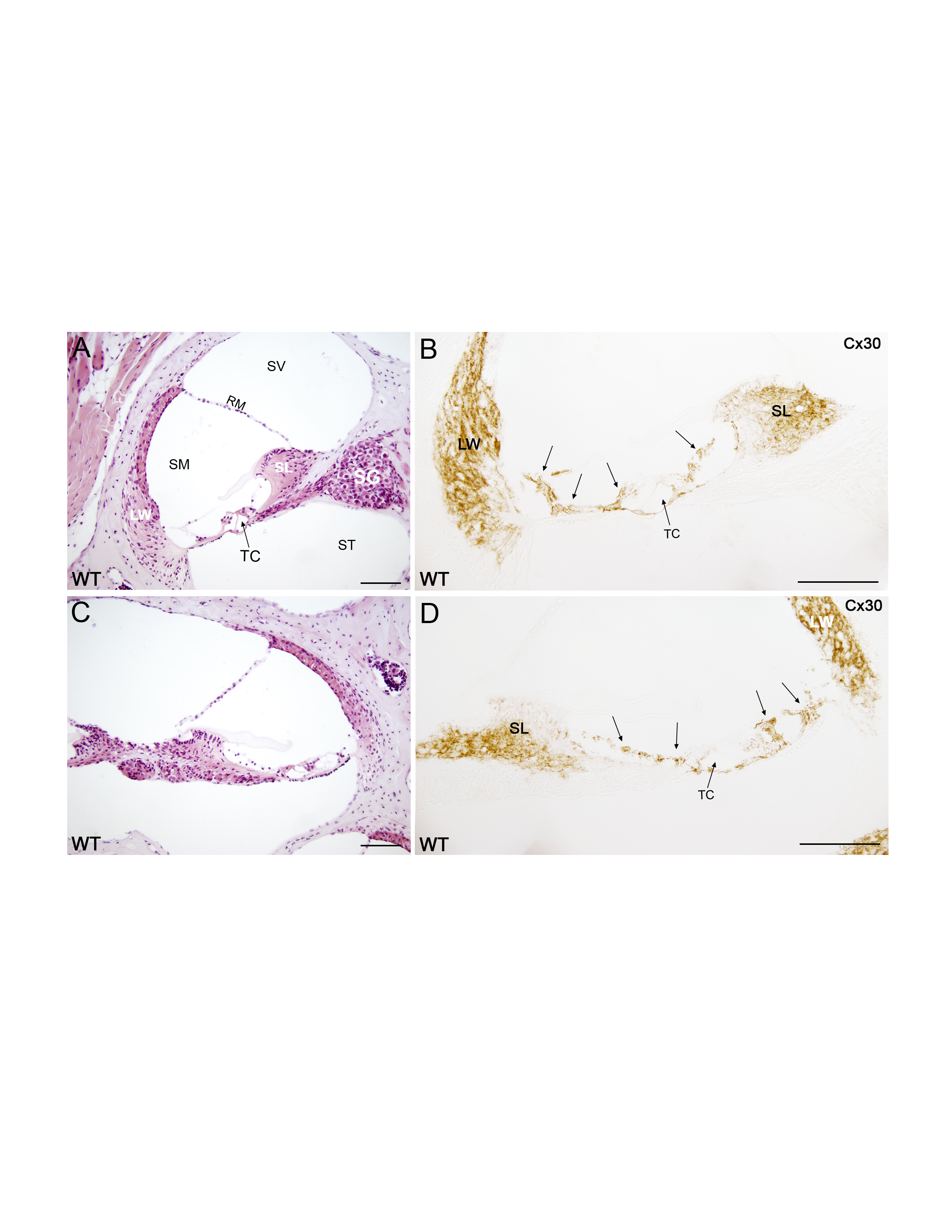


**Suppl.** **Fig. S4. Cx30 immunostaining in the cochlea of P30 WT mice.** Micrographs of paraffin cochlear sections stained with hematoxylin-eosin (**A**, **C**) or immunostained with antibodies against connexin 30, in the middle (**A**, **B**) and apical cochlear turn (**C**, **D**) of WT mice. We observed a normal organ of Corti with an open tunnel of Corti (TC, in **A** and **C**) and abundant Cx30 expression in the spiral limbus, in supporting cells of the organ of Corti (arrows in **B** and **D**) and in the spiral ligament within the lateral wall. LW, lateral wall; RM, Reissner membrane; SG, spiral ganglion; SL, spiral limbus; SM, scala media; ST, scala tympani; SV, scala vestibuli; TC, tunnel of Corti. Scale bar: 100 μm (A, C) and 50 μm (B, D)


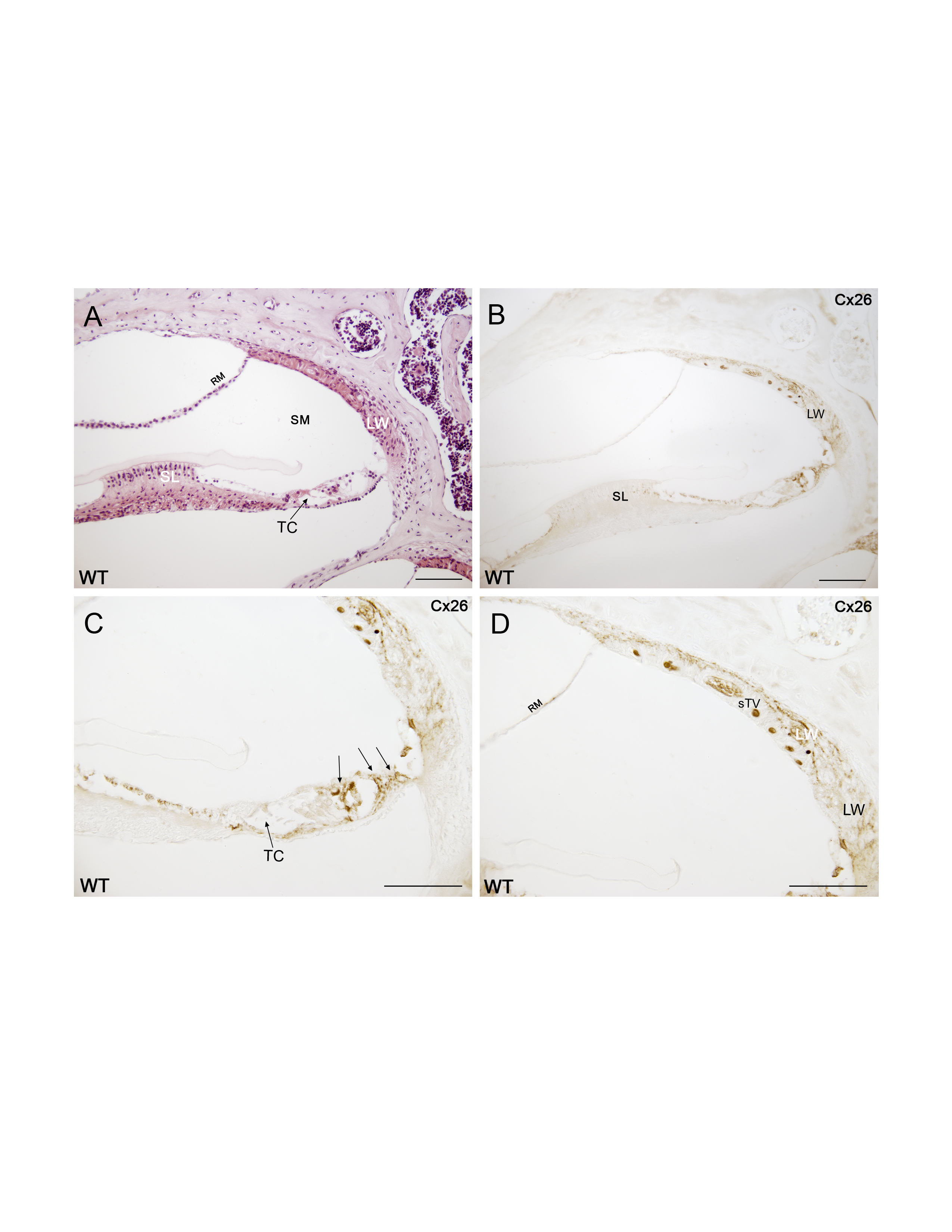


**Suppl. Fig. S5. Cx26 expression in the cochlea of P30 WT mice.**  Micrographs of paraffin cochlear sections stained with hematoxylin-eosin (**A**) or immunostained with antibody against Cx26 (**B-D**), showing the scala media in the apical cochlear turn of WT mice (**A**, **B**) and details of the organ of Corti (**C**) and lateral wall (**D**), with positive Cx26 signal at the spiral limbus, supporting cells of organ of Corti (arrows) and the lateral wall. LW, lateral wall; RM, Reissner membrane; SL, spiral limbus; SM, scala media SL, sTV, stria vascularis; TC, tunnel of Corti. Scale bar: 100 μm (A-B) and 50 μm (C-D)
